# Intestinal Dysbiosis in Autoimmune Diabetes Is Correlated With Poor Glycemic Control and Increased Interleukin-6: A Pilot Study

**DOI:** 10.3389/fimmu.2018.01689

**Published:** 2018-07-25

**Authors:** Bruna Stevanato Higuchi, Nathália Rodrigues, Marina Ignácio Gonzaga, João Carlos Cicogna Paiolo, Nadine Stefanutto, Wellington Pine Omori, Daniel Guariz Pinheiro, João Luiz Brisotti, Euclides Matheucci, Vânia Sammartino Mariano, Gislane Lelis Vilela de Oliveira

**Affiliations:** ^1^Microbiome Study Group, School of Health Sciences Dr. Paulo Prata (FACISB), Barretos, Brazil; ^2^QGene-Solutions and Logistics in Health, Sao Carlos, Brazil; ^3^Board of Health from Barretos, Barretos, Brazil; ^4^Department of Technology, School of Agricultural and Veterinarian Sciences, São Paulo State University (UNESP), Jaboticabal, Sao Paulo, Brazil; ^5^DNA Consult Genetics and Biotechnology, Sao Carlos, Brazil; ^6^Biotechnology Department, Sao Carlos Federal University, UFSCAR, Sao Carlos, Brazil; ^7^Barretos Cancer Hospital (HCB), Barretos, Brazil; ^8^Institute of Biosciences, Humanities and Exact Sciences (IBILCE), São Paulo State University (UNESP), Sao Jose do Rio Preto, Sao Paulo, Brazil

**Keywords:** type 1 diabetes, dietary habits, intestinal dysbiosis, inflammatory cytokines, interleukin-6, glycemic control

## Abstract

Intestinal dysbiosis associated with immunological deregulation, leaky gut, bacterial translocation, and systemic inflammation has been associated with autoimmune diseases, such as type 1 diabetes (T1D). The aim of this study was to investigate the intestinal dysbiosis in T1D patients and correlate these results with clinical parameters and cytokines. The present study was approved by the Barretos Cancer Hospital (Process number 903/2014), and all participants have signed the informed consent in accordance with the Declaration of Helsinki, and answered a questionnaire about dietary habits. Stool samples were used for bacterial 16S sequencing by MiSeq Illumina platform. IL-2, IL-4, IL-6, IL-10, IL-17A, TNF, and IFN-γ plasma concentrations were determined by cytometric bead arrays. The Pearson’s chi-square, Mann–Whitney and Spearman correlation were used for statistical analyses. Alpha and beta diversities were conducted by using an annotated observed taxonomic units table. This study included 20 patients and 28 controls, and we found significant differences (*P* < 0.05) among consumption of vegetables, proteins, milk and derivatives, spicy food, and canned food when we compare patients and controls. We detected intestinal dysbiosis in T1D patients when we performed the beta diversity analysis (*P* = 0.01). The prevalent species found in patients’ stool were the Gram-negatives *Bacteroides vulgatus, Bacteroides rodentium, Prevotella copri*, and *Bacteroides xylanisolvens*. The inflammatory interleukin-6 was significantly increased (*P* = 0.017) in patients’ plasma. Furthermore, we showed correlation among patients with poor glycemic control, represented by high levels of HbA1_C_ percentages and Bacteroidetes, Lactobacillales, and *Bacteroides dorei* relative abundances. We concluded that there are different gut microbiota profiles between T1D patients and healthy controls. The prevalent Gram-negative species in T1D patients could be involved in the leaky gut, bacterial translocation, and poor glycemic control. However, additional studies, with larger cohorts, are required to determine a “signature” of the intestinal microbiota in T1D patients in the Brazilian population.

## Introduction

Type 1 diabetes (T1D) or autoimmune diabetes is a chronic disease mediated by immune reactions against pancreatic beta cells, resulting in insulin dependence to regulate blood glucose concentrations (1). The T1D pathogenesis involves the interaction of genetic and environmental factors, such as viral infections, vitamin deficiencies, and intestinal dysbiosis (2). According to the International Diabetes Federation, more than 96,000 children and adolescents under 15 years will be diagnosed with autoimmune diabetes annually worldwide (3).

The gut microbiota might modulate the T1D pathogenesis *via* two mechanisms. In the first step, an impaired tolerance process in infancy can predispose to develop autoimmune diseases, such as T1D, and might induce autoreactive T cell activation and autoantibodies (4). At the second step, intestinal dysbiosis may lead children with genetic predisposition and positive autoantibodies to develop clinical disease (4).

Studies in animal models reported an increased relative abundance of *Bacteroides, Ruminococcus*, and *Eubacterium* genera in biobreeding diabetes-prone rats, and an increased abundance of *Bifidobacterium* and *Lactobacillus* in diabetes-resistant rats (5). Also, the prevalence of the Bacteroidetes phylum members could promote increased intestinal permeability and precede the clinical onset of T1D in animal models of the disease, in pre-diabetic patients and in diabetic subjects (6, 7).

In T1D children, there is decreased gut microbiota diversity and abundance of mucin-degrading and butyrate-producing members, and reduced Firmicutes/Bacteroidetes ratio, along with *Lactobacillus, Bifidobacterium*, and *Prevotela* species (8). Smaller relative abundance of lactate-producing bacteria, including the *Bifidobacterium longum*, and increased *Clostridium, Bacteroides*, and *Veillonella* species are equally detected (9).

Moreover, intestinal dysbiosis was detected in pre-diabetic children with genetic predisposition and beta cells autoantibodies (10). Increased *Bacteroides dorei* and *Bacteroides vulgatus* species in seroconverted T1D patients are registered 8 months prior to beta cell autoimmunity, suggesting that early dysbiosis may predict T1D in genetically predisposal subjects (11). Additionally, children with beta cell autoantibodies exhibited increased Bacteroidetes members and decreased lactate and butyrate-producing bacteria (12). These studies support the hypothesis that there is a gut microbiome signature associated with T1D development in seropositive children (13).

Previous reports suggested the involvement of the gut mucosa in the pathogenesis of islet autoimmunity in T1D (14). Thus, the modulation of interactions between commensal microbiota and gut-associated lymphoid tissues may represent a means to affect the progression of the autoimmune diabetes (15). Based on previous studies that identify that dysbiosis may be strongly correlated with barrier disruption, bacterial translocation, and autoimmune diseases development (16), we hypothesized that the prevalence of Gram-negative bacteria in the gut mucosa from T1D patients is greater in patients than in stool samples from controls, and positively correlated with poor glycemic control and systemic inflammatory cytokines. In the present study, we investigate the intestinal dysbiosis in T1D patients and correlated these results with clinical data and systemic inflammatory cytokines.

## Materials and Methods

### Patients and Controls Enrollment

Type 1 diabetes patients with fasting blood glucose greater than 126 mg/dL (3) at diagnosis were enrolled by the physician from the endocrinology department from Board of Health from Barretos, Sao Paulo, Brazil, from June 1st, 2015 to July 30th, 2016. A total of 20 patients, 14 females and 6 males (mean age ± SD = 23.1 ± 8.6 years), were included in the present study.

Twenty-eight healthy subjects, 18 females and 10 males (mean age ± SD = 25.2 ± 9.8 years), were enrolled in the present study that was performed in accordance with the recommendations of Ethics committee from Barretos Cancer Hospital. All participants have signed the informed consent in accordance with the Declaration of Helsinki. The present study was approved by the Barretos Cancer Hospital (Process number 903/2014). After the consent, the peripheral blood was collected and stool samples were delivered within 5 days. Stool and plasma samples were stored at −80°C until DNA extraction and cytokine quantification.

At enrollment, all of the subjects answered a questionnaire about dietary habits, such as daily consumption of vegetables, fresh fruits, carbohydrates, proteins (meat/eggs), trans fat, milk and derivatives, spicy food, canned food, hot drinks (coffee/tea), and alcohol. Exclusion criteria include use of anti-inflammatories, antibiotics, laxatives, vaccination, and corticosteroids in the last 30 days. Chronic diarrheas and gastrointestinal surgeries were also considered as exclusion criteria for patients and controls.

Anthropometric measurements and clinical data from T1D patients, such as weight, height, fasting blood glucose and glycated hemoglobin (HbA1c), and disease duration were recorded. The BMI mean was 23.9 ± 3.6 (nine patients presented BMI < 25 and seven patients were overweighed, with BMI > 25). The fasting blood glucose mean was 236.8 ± 135.9 mg/dL (five patients with controlled blood glucose < 126 mg/dL, 11 patients with uncontrolled blood glucose > 126 mg/dL, and four patients without this clinical data). The HbA1_C_ mean was 9.8 ± 1.8% (13 patients with poor glycemic control, HbA1_C_ > 7.5%, one patient with moderate glycemic control, HbA1_C_ = 7%, and six patients without this clinical data). The disease duration mean was 14.0 ± 7.2 years. Demographic characteristics, anthropometric measurements, and clinical data from T1D patients, were summarized in Table 1.

**Table 1:** Demographic, anthropometric, and clinical characteristics obtained from T1D patients.

Patients	Gender/age	Ethnicity	Weight (kg)	Height (m)	Fasting blood glucose (mg/dL)	HbA1_C_ (%)	Disease duration (years)
T1D01	F/28	Caucasian	69	1.64	348.0	9.4	12
T1D02	M/30	Caucasian	84	1.70	221.0	8.2	11
T1D03	M/21	Caucasian	62	1.78	122.0	10.4	14
T1D04	F/24	Caucasian	64	1.73	310.0	10.7	07
T1D05	F/27	Caucasian	70	1.67	283.0	9.6	15
T1D06	M/14	Caucasian	54	1.64	584.0	13.3	02
T1D07	F/18	Caucasian	54	1.58	223.0	10.7	14
T1D08	F/8	Caucasian	32	1.32	88.0	8.9	05
T1D09	F/22	Caucasian	65	1.56	322.0	ND	15
T1D10	M/9	Afrodescendent	38	1.40	132.0	7.5	02
T1D11	F/32	Caucasian	46	1.51	328.0	11.9	29
T1D12	F/12	Afrodescendent	57	1.60	357.0	12.1	02
T1D13	M/30	Caucasian	85	1.70	ND	ND	05
T1D14	F/25	Caucasian	66	1.63	108.0	ND	14
T1D15	F/13	Caucasian	45	1.48	168.0	9.4	ND
T1D16	F/25	Caucasian	85	1.75	ND	ND	12
T1D17	F/28	Caucasian	63	1.60	86.0	8.6	17
T1D18	M/25	Caucasian	93	1.79	110.0	7.0	11
T1D19	F/42	Afrodescendent	71	1.62	ND	ND	09
T1D20	F/30	Caucasian	77	1.73	ND	ND	21

*M, male; F, female; HbA1_C_, glycated hemoglobin (reference: 4.0–6.0%); ND, not determined. Fasting blood glucose near to blood collection (reference < 126 mg/dL)*.

### DNA Extraction and Bacterial 16S Sequencing

**Figure 1:**
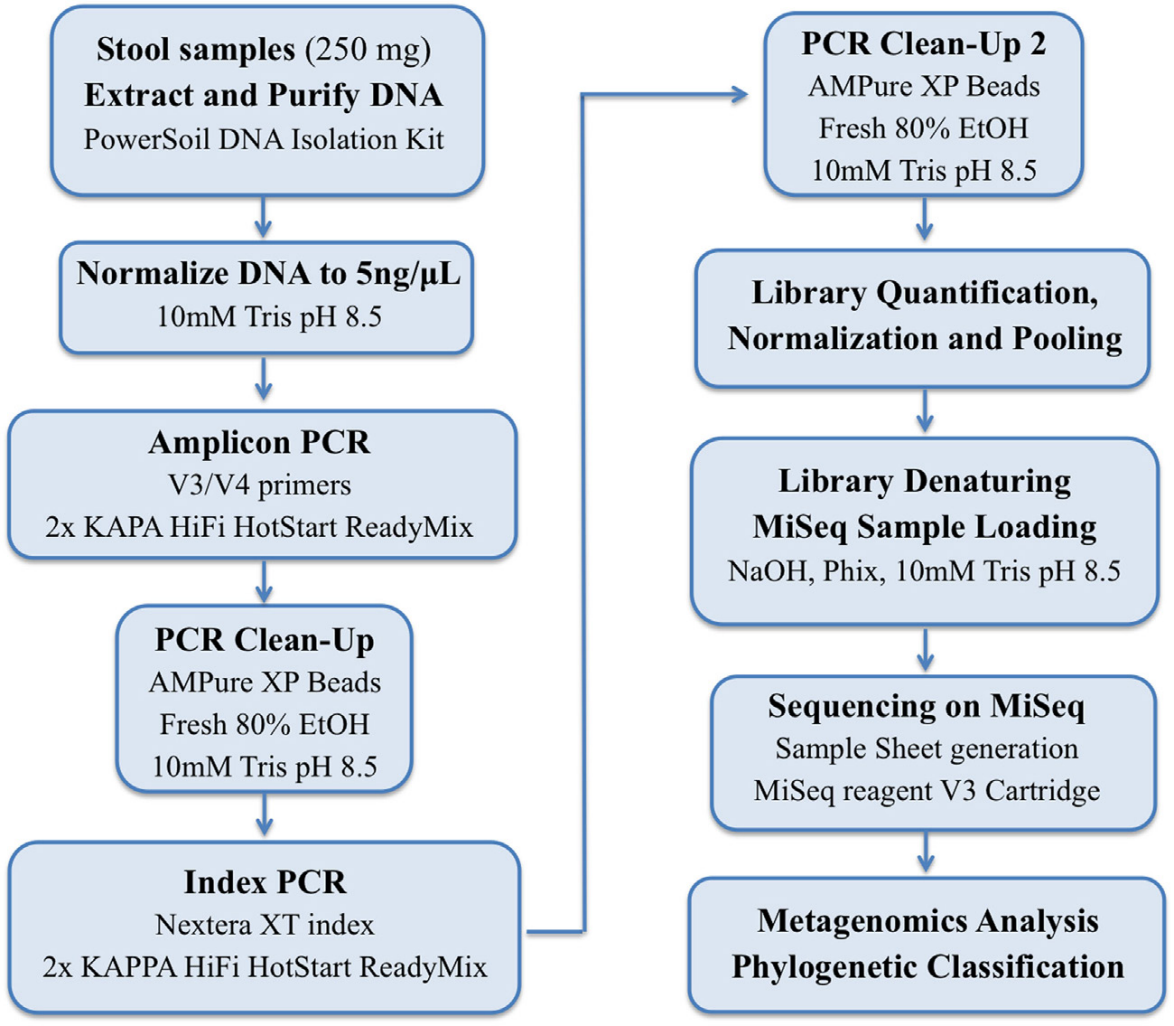
Workflow using the 16S Library Preparation Protocol in Illumina MiSeq platform.

DNA was extracted from 250 mg from stool samples by using PowerSoil DNA Isolation Kit (MO BIO Laboratories, QIAGEN, CA, USA), according to manufacturer’s instructions. DNA quantification was performed by Quantus fluorometer and adjusted to 5 ng/mL with Tris buffer (10 mM, pH 8.5). V3 and V4 regions of the bacterial 16S (17) (Forward primer: 5′-TCGTCGGCAGCGTCAGATGTGTATAAGAGACAGCCTACGGGNGGCWGCAG; Reverse primer: 5′GTCTCGTGGGCTCGGAGATGTGTATAAGAGACAGGACTACHVGGGTATCTAATCC) were amplified by PCR, using bacterial DNA (2.5 mL), primers (5 mL), and 12.5 mL of 2× KAPA HiFi HotStart Ready Mix (Kapa Biosystems, MA, USA). Purification of PCR products was performed by AMPure XP Beads Kit (BD Biosciences, CA, USA). DNA libraries were constructed according to the Illumina protocols, according to the workflow represented in Figure 1. The sequencing was performed by using an Illumina MiSeq platform system (MiSeq Desktop Sequencer).

### Cytokine Determination by Cytometric Bead Array

Peripheral blood (10 mL) was collected from T1D patients and controls and plasma-EDTA was isolated by centrifugation at 1,372 *g*, for 10 min, 4°C. Cytokine detection was performed by cytometric bead array (Human Th1/Th2/Th17 Cytokine Kit, BD Biosciences, CA, USA). Plasma levels of IL-2, IL-4, IL-6, IL-10, IL-17A, TNF, and IFN-γ were determined by flow cytometer FACSCanto™ II (BD Biosciences). Analyses was performed by BDFCAP array™ software and data were expressed by conversion of the median fluorescence intensity in picogram per milliliter.

### Statistical Analyses

Data extracted from the questionnaires containing dietary habits were analyzed by Pearson’s chi-square. Comparisons between cytokines plasma concentrations in T1D patients and controls subjects were performed by Mann–Whitney. Correlations among reads percentages of the intestinal microbiota, cytokines plasma concentrations, and clinical data were performed by Spearman correlation. Analyses of variance, diversity indexes, and alpha and beta diversities were performed by using annotated Operational Taxonomic Units table. Sequencing analyses of the bacterial 16S was performed as described in our previous work (18). *P* values less than 0.05 were considered statistically significant.

## Results

### Dietary Habits and Correlations With the Gut Microbiota in T1D Patients

**Figure 2:**
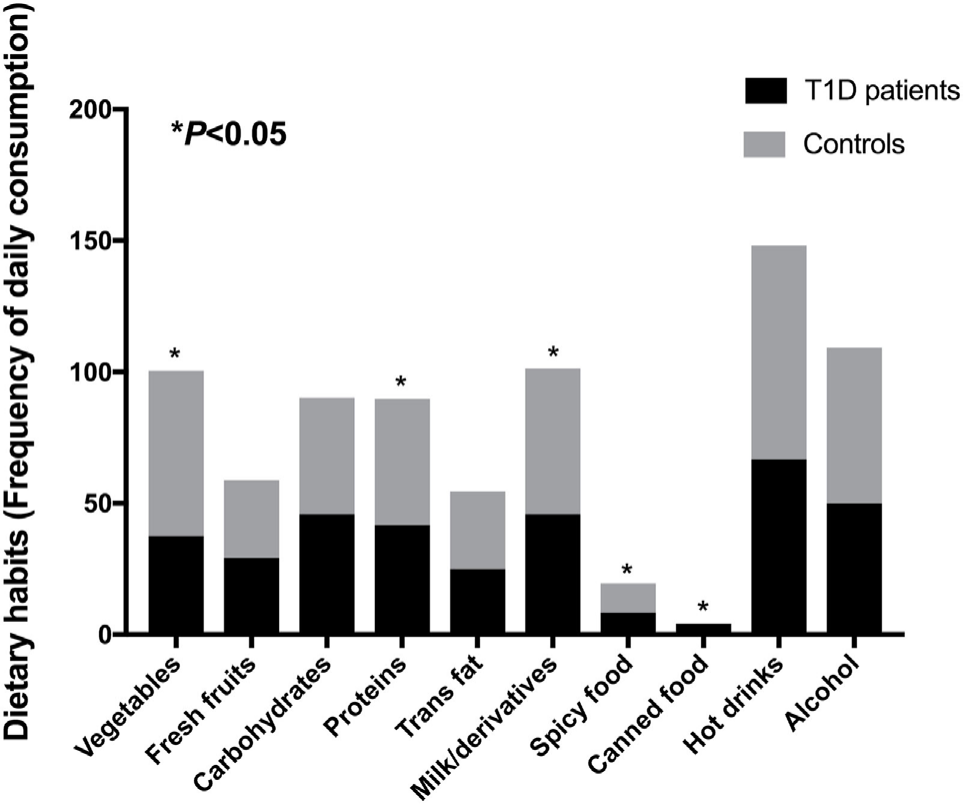
Dietary habits from T1D patients and control subjects. Statistical analyses were performed by Pearson’s chi-square. Significance was set at *P* < 0.05. Horizontal bars represent percentages of positive answer for variables derived from the questionnaire.

To evaluate the dietary habits in T1D patients and healthy controls, we applied a questionnaire about daily consumption of vegetables, fresh fruit, carbohydrates, proteins, trans fat, milk and derivatives, spicy food, canned food, hot drinks (coffee/tea), and alcohol. Regarding these variables, subjects reported daily consumption of vegetables [patients (P) = 37.5%; controls (C) = 63%], fresh fruits [P = 29.2%; C = 29.6%, carbohydrates (P = 45.8%; C = 44.4%), proteins (meat/eggs) (P = 41.7%; C = 48.1%), trans fat (P = 25%; C = 29.6%), milk and derivatives (P = 45.8%; C = 55.6%), spicy food (P = 8.3%; C = 11.1%), canned food (P = 4.2%; C = 0%), hot drinks (P = 66.7%; C = 81.5%), and alcohol (P = 50%; C = 59.3%)]. We observed significant differences (*P* < 0.05) among consumption of vegetables, proteins, milk and derivatives, spicy food, and canned food (Figure 2).

To find correlations between dietary habits and microbial community, we analyzed the correlations among consumption of vegetables, fresh fruit, carbohydrates, proteins, trans fat, milk and derivatives, spicy food, canned food, hot drinks, and alcohol with relative abundances of the gut microbiota detected in stool samples from T1D patients. There were correlations between vegetables intake by patients and Clostridia (*P* = 0.012; *r* = −0.62) and Clostridiales relative abundances (*P* = 0.002; *r* = −0.74), canned food consumption and Bacteroidetes (*P* = 0.002; *r* = 0.73), and *Flavobacterium* (*P* < 0.001; *r* = 0.81) relative abundances. Furthermore, there is an inverse correlation between fresh fruits intake and *Bacteroides* (*P* = 0.016; *r* = −0.59), *B. vulgatus* (*P* = 0.006; *r* = −0.67), and *Bacteroides rodentium* (*P* < 0.001; *r* = −0.81) relative abundances, and between protein intake and Clostridiaceae (*P* = 0.036; *r* = −0.52), *Oscillospira*, and *Oscillospira eae* (*P* = 0.006; *r* = −0.68) relative abundances. We also found correlation between hot drinks intake by patients and *B. rodentium* (*P* = 0.017; *r* = 0.59) abundance, and between milk and derivatives consumption with *Akkermansia muciniphila* (*P* = 0.016; *r* = −0.59) relative abundance.

### Detection of Intestinal Dysbiosis in T1D Patients

**Figure 3:**
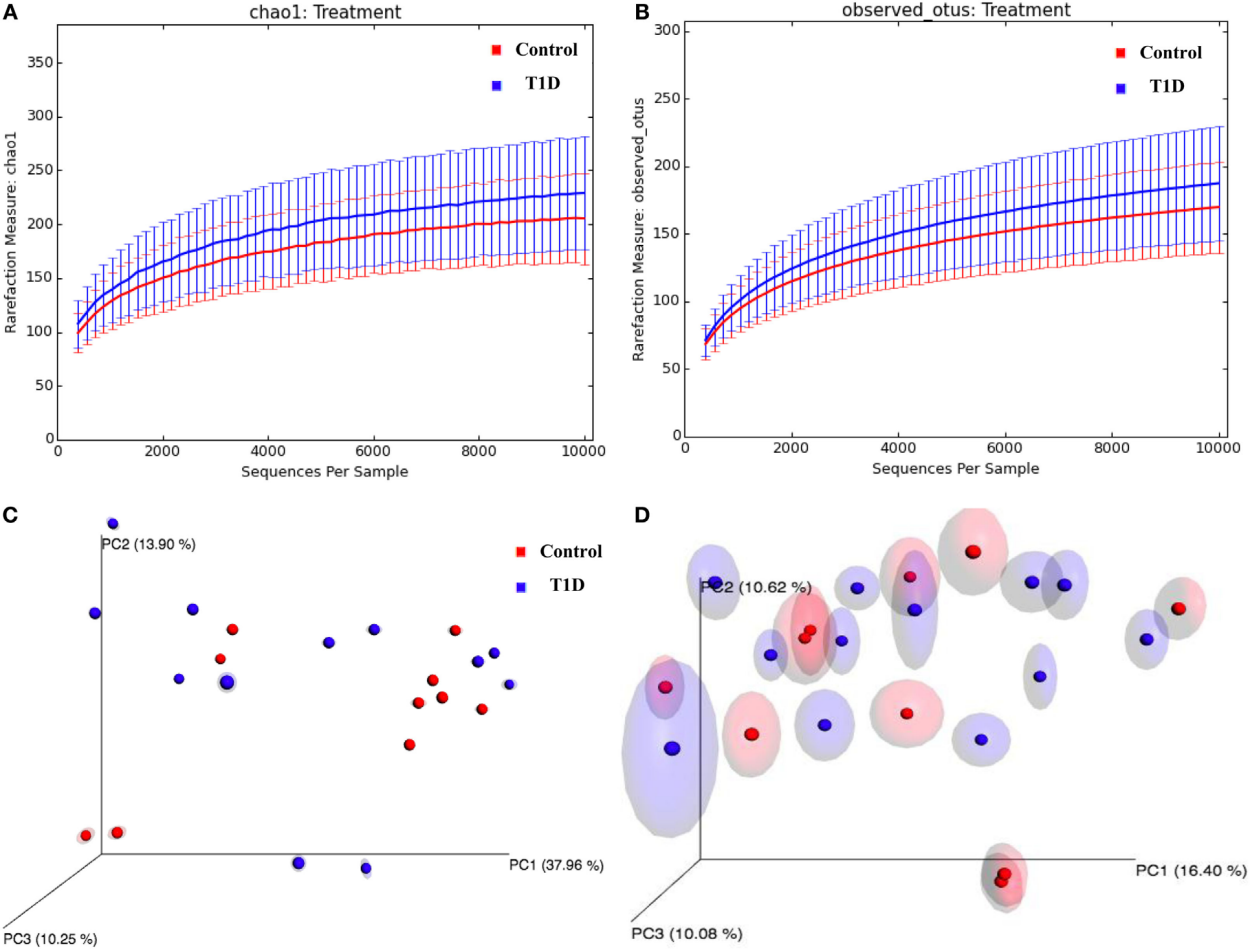
Alpha and beta-diversity in gut microbiota from T1D patients. **(A,B)** Rarefaction curves comparing the species richness, Chao1, and OTU numbers. **(C,D)** PcoA plots with weighted and unweighted UniFrac metric with Bonferroni’s correction.

To investigate the dysbiosis in T1D patients, we sequenced the 16S bacterial DNA stool samples from patients and controls and calculated alpha and beta diversities. According to the rarefaction curves, there were no significant differences (*P* = 0.318) in richness and evenness between samples obtained for patients and controls (Figures 3A,B; Table 2). However, we detected significant differences (*P* = 0.01) between microbial community found in T1D patients and controls, when we performed the weighted and unweighted UniFrac metric with Bonferroni’s correction analyses (Figures 3C,D).

**Table 2:** Diversity and richness index results from alpha-diversity analyses in T1D patients.

Sample ID	Simpson	Shannon	Chao1	Observed OTUs
T1D02	0.921	5.078	339.65	252
T1D05	0.935	5.283	292.11	246
T1D06	0.932	4.948	216.11	150
T1D07	0.949	5.216	202.64	176
T1D08	0.950	5.053	148.42	127
T1D09	0.848	4.666	237.05	200
T1D11	0.943	5.087	238.93	201
T1D14	0.958	5.667	262.53	231
T1D16	0.943	5.049	164.15	152
T1D17	0.964	5.900	277.00	238
T1D18	0.915	4.609	194.00	142
T1D19	0.956	5.309	199.15	156
CTL02	0.931	4.791	202.57	155
CTL05	0.955	5.282	212.26	173
CTL08	0.924	4.934	148.25	134
CTL16	0.945	5.161	169.00	159
CTL21	0.950	4.930	145.06	118
CTL23	0.934	5.220	214.00	177
CTL26	0.930	5.117	279.71	209
CTL30	0.955	5.179	192.40	148
CTL38	0.971	5.923	251.54	220

### Prevalence of Gram-Negatives in the Gut Microbiota in T1D Patients

**Figure 4:**
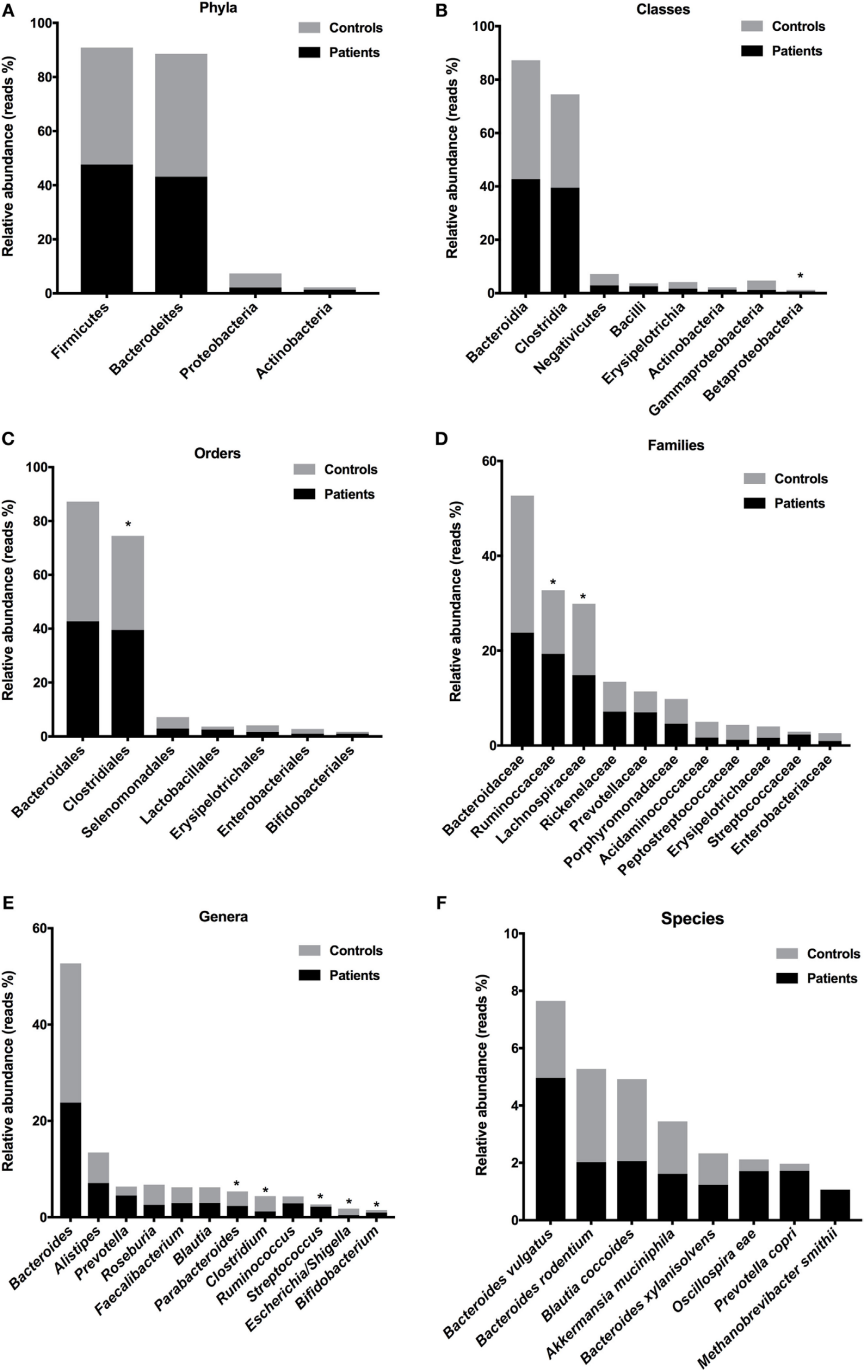
Relative abundances of bacterial taxa in stool samples from T1D patients. Predominant phyla **(A)**, classes **(B)**, orders **(C)**, families **(D)**, genera **(E)**, and species **(F)**. Bars represent the reads percentages found in metagenomics analyses. *P < 0.05.

To evaluate microbiota composition in stool samples from patients and controls, we compare the reads percentages in both groups. The prevalent phyla in T1D patients were Firmicutes [patient reads (PR) = 47.62%; control reads (CR) = 43.23%] and Bacteroidetes (PR = 43.13%; CR = 45.44%), the prevalent classes were Bacteroidia (PR = 42.73%; CR = 44.52%) and Clostridia (PR = 39.50%; CR = 35.01%), the prevalent orders were Bacteroidales (PR = 42.73%; CR = 44.52%) and Clostridiales (PR = 39.50%; CR = 35.00%), and the prevalent families were Bacteroidaceae (PR = 23.78%; CR = 28.91%), Ruminococcaceae (PR = 19.32%; CR = 13.42%), and Lachnospiraceae (PR = 14.83%; CR = 15.04%) (Figures 4A–D). The prevalent genera in T1D patients were *Bacteroides* (PR = 23.78%; CR = 28.91%), *Alistipes* (PR = 7.12%; CR = 6.32%), and *Prevotella* (PR = 4.50%; CR = 1.89%). The prevalent species in the feces of T1D patients were *B. vulgatus* (PR = 4.96%; CR = 2.69%), *Blautia coccoides* (PR = 2.06%; CR = 2.85%), *B. rodentium* (PR = 2.03%; CR = 3.25%), *Prevotella copri* (PR = 1.72%; CR = 0.25%), *Oscillospira eae* (PR = 1.71%; CR = 0.41%), *A. muciniphila* (PR = 1.61%; CR = 1.82%), and *Bacteroides xylanisolvens* (PR = 1.23%; CR = 1.09%) (Figures 4E,F). Additionally, we detected significant differences in relative abundance of Betaproteobacteria class (*P* = 0.021), Clostridiales order (*P* = 0.007), Ruminococcaceae (*P* = 0.027) and Lachnospiraceae (*P* = 0.008) families, and in *Escherichia/Shiguela* (*P* = 0.005), *Bifidobacterium* (*P* = 0.021), *Parabacteroides* (*P* = 0.021), *Streptococcus* (*P* = 0.033), *Bacteroides* (*P* = 0.038), and *Clostridium* (*P* = 0.043) genera (Figure 4).

### Intestinal Dysbiosis Correlated With Poor Glycemic Control

**Figure 5:**
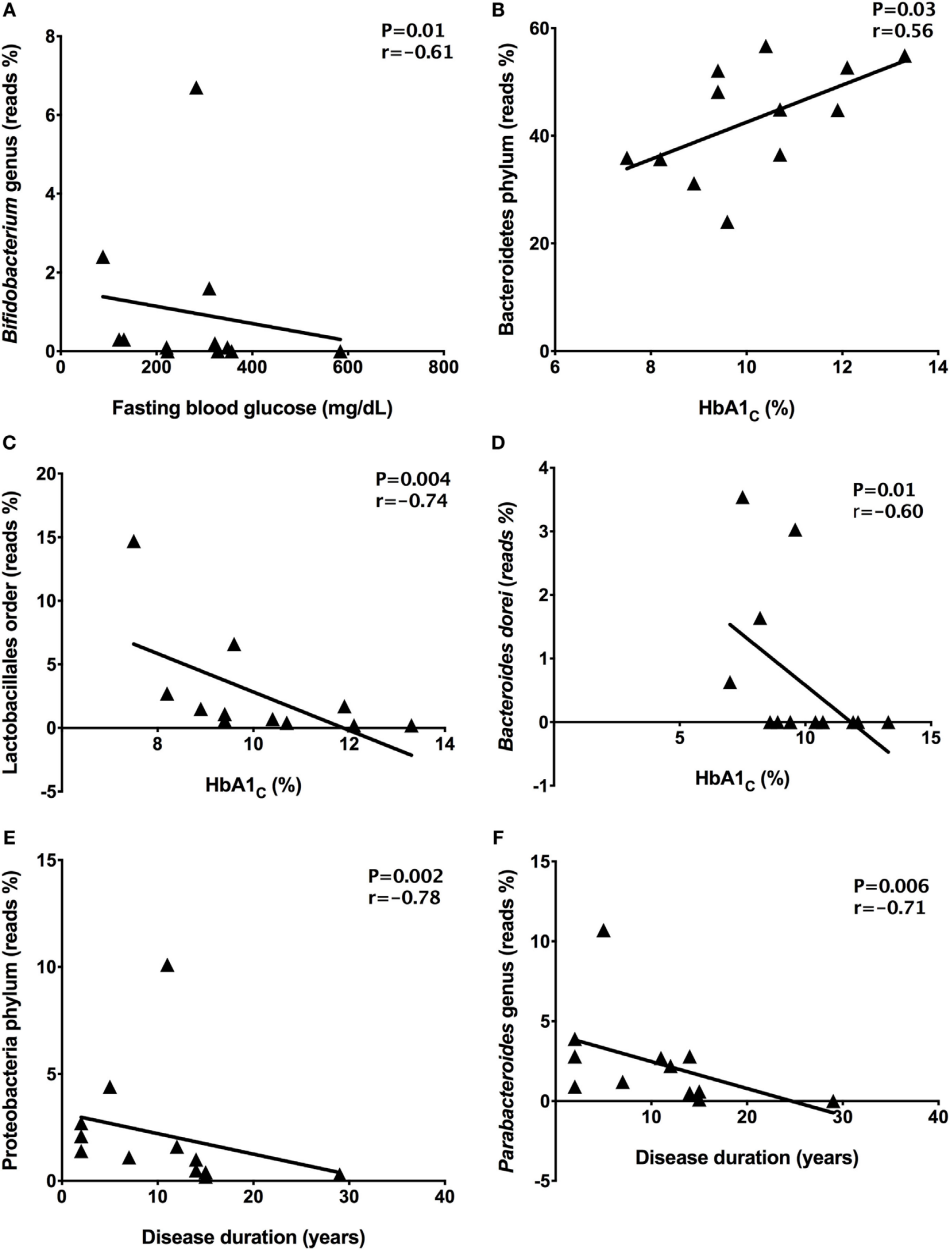
Correlations among relative abundance of bacterial community and clinical data. *Bifidobacterium* and fasting blood glucose **(A)**, Bacteroidetes and HbA1_C_ **(B)**, Lactobacillales and HbA1_C_ **(C)**, *Bacteroides dorei* and HbA1_C_ **(D)**, Proteobacteria and disease duration **(E)**, *Parabacteroides* and disease duration **(F)**. Statistical analyses were performed by using the Spearman’s test. Significance was set at *P* < 0.05.

To find correlations between microbial community and clinical results, we analyzed the correlations among fasting blood glucose, HbA1c, and disease duration with relative abundances of the gut microbiota detected in stool samples from T1D patients. There was a negative correlation (*P* = 0.01, *r* = −0.61) between *Bifidobacterium* members with fasting blood glucose in T1D patients (Figure 5A). Moreover, correlations among high levels of HbA1_C_ percentages and Bacteroidetes (*P* = 0.03, *r* = 0.56), Lactobacillales (*P* = 0.004, *r* = −0.74), and *Bacteroides dorei* relative abundances (*P* = 0.01, *r* = −0.60) were detected (Figures 5B–D). Additionally, there was a negative correlation among disease duration and Proteobacteria (*P* = 0.002, *r* = −0.78) and *Parabacteroides* members (*P* = 0.006, *r* = −0.71) (Figures 5E,F).

### Inflammatory Cytokines Positively Correlated With Gram-Negative Bacterial Community

**Figure 6:**
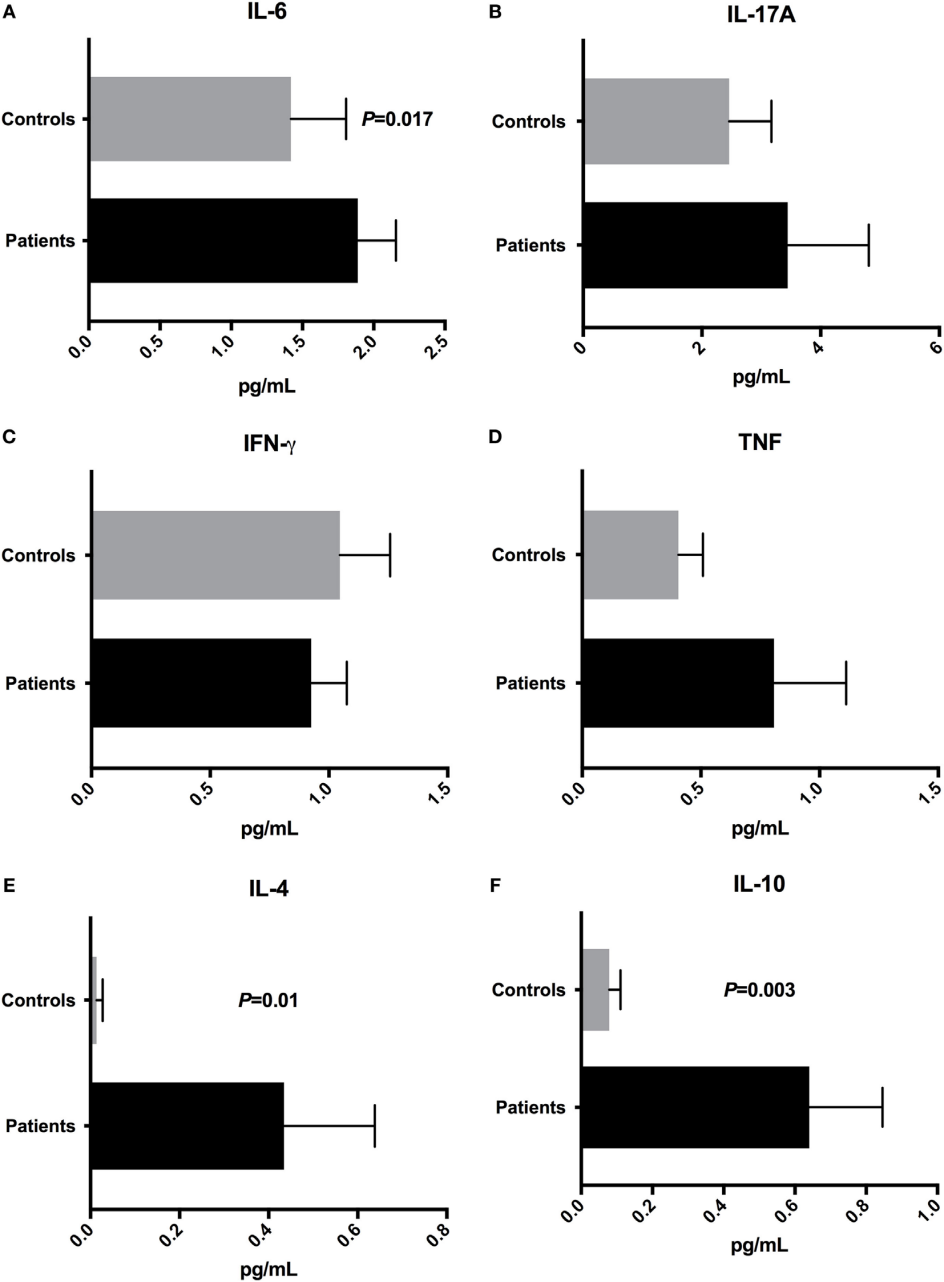
Cytokine profile in T1D patients and control subjects. Plasma concentrations of **(A)** IL-6, **(B)** IL-17A, **(C)** IFN-γ, **(D)** TNF, **(E)** IL-4, and **(F)** IL-10. Statistical analyses were performed by Mann–Whitney test. Significance was set at *P* < 0.05.

To determine the plasma concentrations of cytokines, we quantified the concentrations of IL-2, IL-4, IL-6, IL-10, IL-17A, IFN-γ, and TNF cytokines by cytometric bead array. IL-2 concentration was undetectable in control samples, and it was impossible to compare with plasma levels obtained for patients (0.308 ± 0.214 pg/mL) (data not shown). The IL-6 plasma concentration was increased (*P* = 0.017) in patients (1.889 ± 0.268 pg/mL) when compared with control group (1.419 ± 0.387 pg/mL) (Figure 6A). Moreover, there were no significant differences (*P* > 0.05) in the concentrations of IL-17A, IFN-γ, and TNF inflammatory cytokines in patients’ plasma (IL-17A: 3.445 ± 1.367 pg/mL; IFN-γ: 0.926 ± 0.15 pg/mL; TNF: 0.808 ± 0.304 pg/mL) compared with controls (IL-17A: 2.459 ± 0.713 pg/mL; IFN-γ: 1.046 ± 0.211 pg/mL; TNF: 0.406 ± 0.103 pg/mL) (Figures 6B–D). The IL-4 (*P* = 0.01) and IL-10 (*P* = 0.003) plasma concentrations were augmented in patients’ samples (IL-4: 0.434 ± 0.204 pg/mL; IL-10: 0.64 ± 0.206 pg/mL), when compared with healthy subjects (IL-4: 0.014 ± 0.013 pg/mL; IL-10: 0.079 ± 0.032 pg/mL) (Figures 6E,F).

**Figure 7:**
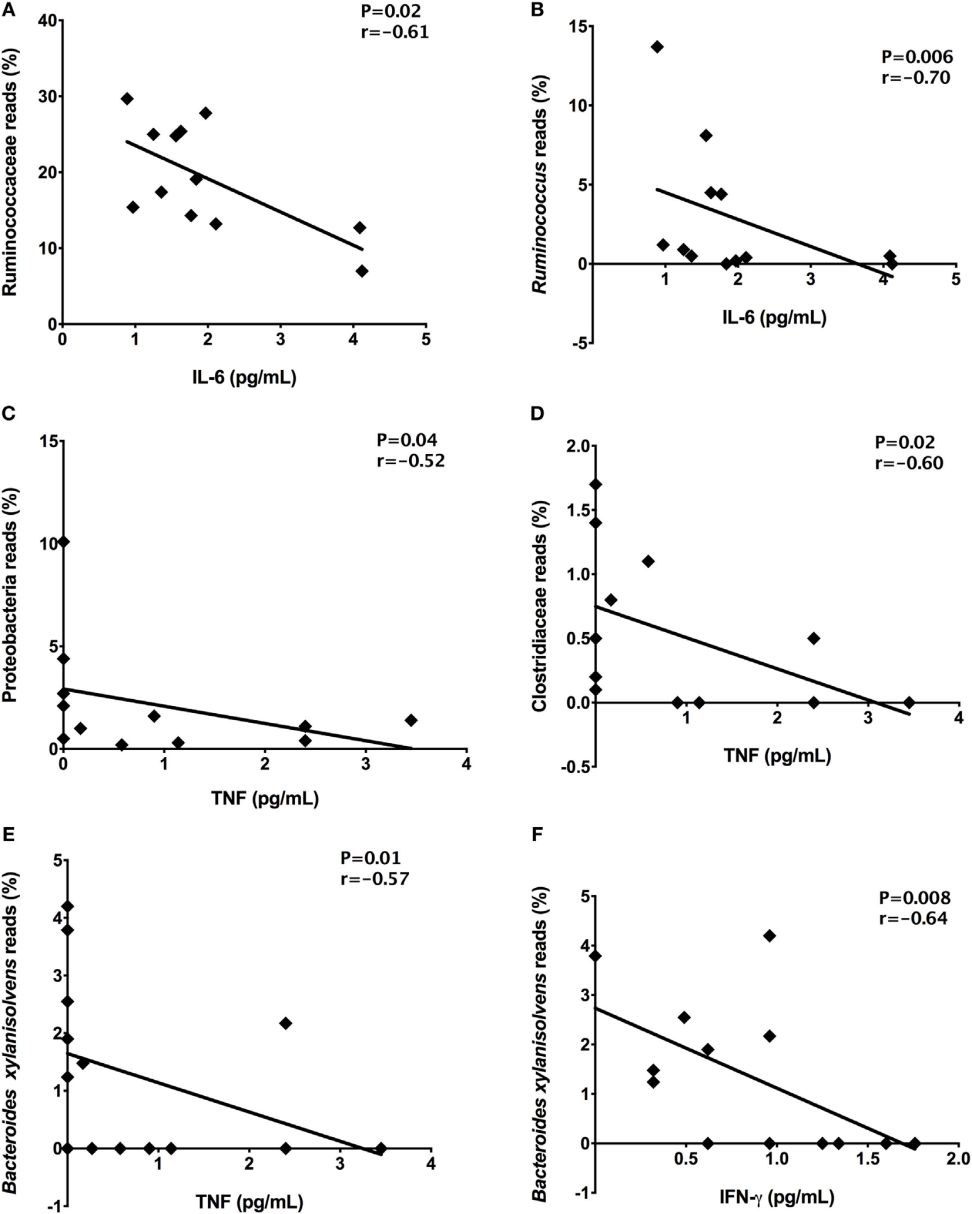
Correlations among the relative abundances of bacterial taxa and plasma concentrations of cytokines. Ruminococcaceae and IL-6 **(A)**, *Ruminococcus* and IL-6 **(B)**, Proteobacteria and TNF **(C)**, Clostridiaceae and TNF **(D)**, *Bacteroides xylanisolvens* and TNF **(E)**, *Bacteroides xylanisolvens and IFN-γ* **(F)**. Statistical analyses were performed by Spearman’s test. Significance was set at *P* < 0.05.

In order to detect correlations between dysbiosis and cytokines, we identified correlations between systemic levels of cytokines and reads percentages of bacterial groups present in stool samples from T1D patients. Significant correlations among the inflammatory cytokine IL-6 and Ruminococcaceae (*P* = 0.02, *r* = −0.61) and *Ruminococcus* members (*P* = 0.006, *r* = −0.70) was detected (Figures 7A,B). The TNF plasma levels negatively correlated with Proteobacteria (*P* = 0.04, *r* = −0.52) and Clostridiaceae relative abundances (*P* = 0.02, *r* = −0.60) (Figures 7C,D). Finally, the IFN-γ (*P* = 0.008, *r* = −0.64) and TNF (*P* = 0.001, *r* = −0.57) plasma concentrations correlated with relative abundances of *B. xylanisolvens* (Figures 7E,F).

## Discussion

The gastrointestinal tract hosts approximately 100 trillions of bacteria that reside in mucosal surfaces and constantly interact with immune cells (14). This microbial community function as microbiological defense barrier, induce antimicrobial peptides secretion and immunological responses that increase mucosal and systemic immunity (19). Previous studies have focused on the role of commensal microbiota in health maintenance and disease development and environmental factors that influence its dynamics (20, 21). The equilibrium between commensal microbiota and host is characterized by microbiota members that improve metabolism and protect against pathobionts and gut inflammation (22). Several evidences suggest that alterations in function and diversity of the gut microbiota might be linked to the development of autoimmune diseases, including T1D (23). The hypotheses proposed to correlate dysbiosis with autoimmune diseases include bystander T-cell activation, molecular mimicry, amplification of autoimmunity by inflammatory milleu, induced by dysbiotic microbiota, and recently proposed, the posttranslational modification of luminal proteins by enzymes from dysbiotic microbiota (24). This altered posttranslational modification of luminal proteins could produce neo-epitopes that may become immunogenic and trigger systemic autoimmunity and autoimmune diseases (24).

Recent reports have demonstrated the influence of dietary habits in the gut microbiota composition (25–28). In the study from Wu and coworkers (2011), they evaluated the association between diet and gut microbiota composition, in a cohort of 98 healthy subjects. Authors reported that the *Bacteroides* genera was associated with animal protein and saturated fat consumption, while *Prevotella* genera was linked to carbohydrates and simple sugar intake (27).

In another study, Yamaguchi and colleagues evaluated dietary habits, metabolic markers, and fecal microbiota in 59 T2D patients. In this study, they reported a correlation between high carbohydrates, fat, and protein consumption with increased counts of *Clostridium* clusters IV and XI. *Bifidobacterium* species, *Lactobacillales*, and *Bacteroides* species were inversely correlated with carbohydrate, protein consumption, and fasting blood glucose, respectively. Authors concluded that low consumption of proteins and carbohydrates favors a healthy gut microbiota and improve glucose tolerance in T2D patients (28). In our work, we detected an inverse correlation between fresh fruits intake and *Bacteroides, B. vulgatus*, and *B. rodentium* abundances, and between protein consumption with Clostridiaceae, *Oscillospira*, and *Oscillospira eae* relative abundances.

In the present study, we reported an intestinal dysbiosis in T1D patients, with significant differences in the diversity of the gut microbiota, represented by beta-diversity analysis between patients and controls. Study from Murri and colleagues investigated the gut microbiota in 16 T1D children and 16 control subjects and demonstrated an increased numbers of *Clostridium, Bacteroides*, and *Veillonella* genera in patients when compared with control counterparts. Furthermore, the Firmicutes/Bacteroidetes ratio and relative abundances of *Lactobacillus, Bifidobacterium*, and *Prevotela* members were decreased in T1D patients (9). Moreover, authors showed correlations among *Lactobacillus, Bifidobacterium*, and *Clostridium* members with glucose plasma levels (9).

In this study, we detected the prevalence of gram-negative species in stool samples from our T1D patients, including *B. vulgatus, B. rodentium, P. copri*, and *B. xylanisolvens*, supporting our hypothesis and suggesting an increase in the bacterial translocation through the epithelial barrier, triggering systemic inflammation, increased oxidative stress, metabolic deregulation, and beta cell destruction (14). Previous studies in animal models showed that gut microbiota translocation to pancreatic lymph nodes triggers NOD2 activation, Th1 and Th17 differentiation, which contribute to inflammatory infiltrate inside the pancreatic islets and T1D development (29).

The adult healthy intestinal microbiota is composed by Firmicutes, Gram-positive members, and Bacteroidetes, Gram-negative members (30). The prevalent species from Firmicutes phylum are *Faecalibacterium prausnitzii* and *Eubacterium rectale/Roseburia* species (31). These bacteria produce short-chain fatty acids, including the butyrate, which suppress NF-κB signaling in the intestinal epithelial cells (31, 32). The Bacteroidetes phylum, the second most prevalent in the human intestine, is dominated by *Bacteroides* and *Prevotella* species (33). The *Prevotella* members are able to activate TLR2 receptors and induce Th17 CD4 T cell differentiation. The prevalence of *Bacteroides* and *Prevotella* species is associated with gastrointestinal inflammation, triggered mainly by the inflammatory Th17 cytokines (34). Additionally, *Prevotella* species induce IL-8 and IL-6 release by epithelial cells, inducing Th17 immune responses and neutrophil recruitment (34). Thus, inflammation of the gastrointestinal mucosa, induced by *Prevotella* species might promote dissemination of inflammatory mediators, barrier dysfunction, and bacterial translocation, which induce and amplify the systemic inflammation (34).

Previous reports showed that T1D children with autoantibodies have an increase in the Gram-negative Bacteroidetes members, reduction in mucin-degradation and butyrate-producing species (10, 35, 36). Butyrate has an anti-inflammatory effect, induces T regulatory cells in the gut mucosa, and enhances the barrier *via* tight-junctions expression (37, 38). In addition, researchers demonstrated that T1D children exhibit diminished abundance of lactate-producing bacteria, including *B. longum*, subspecies *infantis*. *Bifidobacterium* species can exert several functions, such as carbohydrate fermentation, acetate and lactate generation, polyphenols and linoleic acids release, and antioxidant activities (39). They also play an important role in the gastrointestinal lymphoid tissue maturation in early life and offer protection against pathogens by bacteriocin release, decrease in luminal pH, and inhibition of epithelial adhesion (40). In agreement with these studies, we detected the prevalence of Gram-negative bacteria in stool samples from our T1D patients and decreased butyrate-producing bacteria, such as *Bifidobacterium* and *Roseburia* species, and decreased *Clostridium* members that could induce anti-inflammatory IL-10 cytokine and T regulatory cells (41).

The germ-free NOD mice have a similar metabolite profile to that of pre-diabetic children, with reduced glycemic control and deregulated immunologic and metabolic responses (42). The absence of a gut microbiota in this mice did not affect the diabetes incidence but promoted insulitis and increased levels of pro-inflammatory IFN-γ and IL-12, suggesting the important role of the microbiota in glucose metabolism (42). Our data suggest that T1D patients with intestinal dysbiosis, with decreased abundance of beneficial microbes, such as *Bifidobacterium* and Lactobacillales members have poor glycemic control, represented by high levels of HbA1c. The standard method to monitor glycemic control is the measurements of the HbA1c, and the good glycemic control is related to decreased microvascular complications in T1D patients (3, 43, 44). The poor glycemic control is defined as HbA1c between 59 and 106 mmol/mol or 7.5–12% (43). About 25% of T1D patients’ population is in persistent poor glycemic control (44), which is in accordance with our present study, that 13 patients (65%) presented poor glycemic control. Our hypothesis relies on that intestinal dysbiosis and bacterial translocation can induce systemic inflammation and suppression of insulin receptors, promoting an increased glucose blood levels and high levels of HbA1c in T1D patients. Gram-negative-derived LPS binds to TLR4 and trigger the inflammatory cascade, resulting in NF-κB activation and secretion of inflammatory mediators, such as TNF, IL-1, and IL-6, which influence glucose metabolism and inhibit phosphorylation of insulin receptors (45–47).

In the present study, T1D patients presented increased inflammatory IL-6 concentrations that inversely correlated with Ruminococcaceae family, an anaerobe microorganism that degrade complex carbohydrates, and are common in subjects with carbohydrate-enriched diets (48). The inflammatory TNF inversely correlated with Clostridiaceae members that include *Clostridium* species, which can induce T regulatory cells in the gut mucosa. The *Clostridium* species, especially clusters IV and XIVa, are spore-forming members from the gut microbiota and can induce T regulatory cells differentiation in the gut mucosa (41).

Proposed mechanisms to explain the link between intestinal dysbiosis and T1D include the dysbiosis-associated immunological deregulation, leading to destruction of β-cells by autoreactive T cells (14, 49). The second proposed mechanism correlates T1D with leaky gut, bacterial translocation, endotoxemia, and chronic low-grade inflammation (12, 50). The Gram-negative bacterial species, prevalent in stool samples from our T1D patients, enhance gut permeability and increase bacterial translocation, which can lead to systemic inflammation and metabolic deregulation (14, 50).

Finally, we concluded that there are different gut microbiota profiles between T1D patients and healthy controls. The prevalent Gram-negative species in T1D patients could be involved in the leaky gut, bacterial translocation, and poor glycemic control. However, additional studies, with larger cohorts, are required to determine a “signature” of the intestinal microbiota in T1D patients in the Brazilian population.

## Ethics Statement

The present study was performed in accordance with the recommendations of Ethics committee from Barretos Cancer Hospital. All subjects provided written informed consent in accordance with the Declaration of Helsinki. The protocol was approved by the Barretos Cancer Hospital (Process number 903/2014).

## Author Contributions

BH: T1D patients’ enrollment, DNA extraction, cytokine determination, data acquisition, and manuscript writing; MG: controls’ enrollment and sample collection; NR and NS: V3/V4 amplification, library construction, and sequencing; JB: support for blood samples collection; EM: support to Illumina platform sequencing; JP: responsible for clinical data from T1D patients. WO and DP: bioinformatics analyses; VM: sample collection, DNA quantification, and cytokine determination; GO: experimental design, data interpretation, manuscript writing, and revision.

## Conflict of Interest Statement

The authors declare that the research was conducted in the absence of any commercial or financial relationships that could be construed as a potential conflict of interest.
